# A Novel Modified Surgical Approach for FIL SSF Lens

**DOI:** 10.7759/cureus.49857

**Published:** 2023-12-03

**Authors:** Georgios Batsos, Nikolaos Bouratzis, Loukas Kontomichos, Diego Ruiz Casas, Spyros Atzamoglou, Vasileios Peponis, Dimitris Karagiannis, Efstratios Paroikakis

**Affiliations:** 1 First Department of Ophthalmology, Specialized Eye Hospital, Ophthalmiatreio Athinon, Athens, GRC; 2 Department of Ophthalmology, Ramon y Cajal Hospital, Madrid, ESP; 3 Second Department of Ophthalmology, Specialized Eye Hospital, Ophthalmiatreio Athinon, Athens, GRC

**Keywords:** scleral-fixated iol, pars plana vitrectomy (ppv), surgical flaps, lens subluxation, aphakia

## Abstract

This study aimed to describe a novel modified surgical technique for FIL SSF lens (Rome, Italy: Soleko) implantation. A retrospective study of FIL SSF lens implantation on six eyes of six patients with subluxated or dislocated intraocular lens (IOL). Standard pars plana vitrectomy (PPV) was performed in all patients. The subluxated or dislocated IOL was removed from a 2.4 corneal incision. From the same incision, the folded FIL SSF lens was inserted. Then lens plugs were extremized through a 23G scleral incision inside two 4 mm pockets that were created at the beginning of the operation. In two cases one pocket had to be converted into a triagonal-shaped scleral flap. All scleral pockets were sutured with 7.0 Vicryl suture and the conjunctiva with 7.0 Vicryl. In the follow-up period of six months, the lens is centered and not tilted. The refractive outcome is within the expectations. Visual acuity is improved in all patients. No haptic exposure and no other complications were noted in all cases. FIL SSF lens is a good option for treating aphakia. This modified implantation technique is safe, fast, and easy. It is also versatile, combining the advantages of both previously described techniques, as it gives the option of flap conversion if needed. Larger studies and prospective comparative studies can highlight the best and more appropriate technique.

## Introduction

The management of aphakia in cases of absent zonular support was always challenging [1]. There are several solutions in the management of such cases, such as the use of anterior chamber (AC) lens, iris claw lenses, or scleral intraocular lens (IOL) fixation. Regarding the scleral lens fixation, several techniques have been described, including suturing the lens into the sclera (e.g., Akreos lens {Rochester, NY: Bausch + Lomb} with GORE-TEX suture {Newark, Delaware: Gore Medical}) [2], or fixating the haptics of a three-piece lens into scleral tunnels [3]. The FIL SSF lens (Rome, Italy: Soleko) is currently the only intraocular lens specially designed for sutureless scleral lens fixation. This hydrophilic foldable lens has a 6.5 mm optic plate and a total diameter of 13.2 mm with two haptics. Each haptic has a T-shaped plug that serves as an intrascleral anchor. For the implantation of the lens, two major techniques have been proposed [4]. The first technique comprises the creation of two scleral 4 mm flaps for covering the two scleral plugs [5]. In the second technique which is less traumatic and less time-consuming, each plug is inserted into two scleral pockets (two pockets on each side) [6]. However, with the latter technique, there are some reports of post-operative hypotony (less watertight), rotation of the haptic, and most importantly, plug exposure under the conjunctiva [4]. A third transconjunctival technique has also been described [7]. The purpose of this study was to describe a novel and more simplified technique for the implantation of these lenses using only two closed scleral rectangular pockets. This technique makes surgery faster and easier and improves scaring, avoiding plug extrusion because the scleral vascular flow is not severed.

## Technical report

This study is adherent to the Declaration of Helsinki. Informed consent has been obtained from all patients before the operation. All patients had subluxated or dislocated IOL into the posterior chamber. In all cases, no other ocular comorbidity from the anterior or posterior segment was present.

Complete ophthalmic examination was performed on all patients, which included best corrected visual acuity measurement estimated using log minimum angle of resolution (logMAR) charts, intraocular pressure measurement, fundoscopy, and optical coherence tomography (OCT) examination. The mean (standard deviation) pre-operative uncorrected logMAR visual acuity was 2.14 (0.15) and was increased to 0.04 (0.03) one month after the operation. Differences in pre-operative and post-operative visual acuities were statistically significant based on the Wilcoxon signed-rank test (p=0.035). Biometry was performed using ZEISS IOLMaster 700 (Jena, Germany: Carl Zeiss Meditec AG). All patients were operated from the same surgeon and re-examined on the first and 10th postoperative days, and then after one, three, and six months. Table 1 summarizes all patients' demographics as well as the type of IOL dislocation and pre-operative visual acuity.

**Table 1:** Baseline characteristics: patients' age/gender, type of dislocation, and pre-operative visual acuity. M: male; F: female; pre-op VA: pre-operative uncorrected visual acuity; MAR: minimum angle of resolution; IOL: intraocular lens

	Age/gender	Type of IOL dislocation	Pre-op uncorrected VA (logMAR)
Patient 1	65/F	Decentration	2.20
Patient 2	73/M	Subluxation	2.25
Patient 3	70/M	Subluxation	2.3
Patient 4	71/M	Dislocation	2.2
Patient 5	80/F	Dislocation	1.9
Patient 6	73/M	Subluxation	1.9

Surgical technique

**Figure 1:**
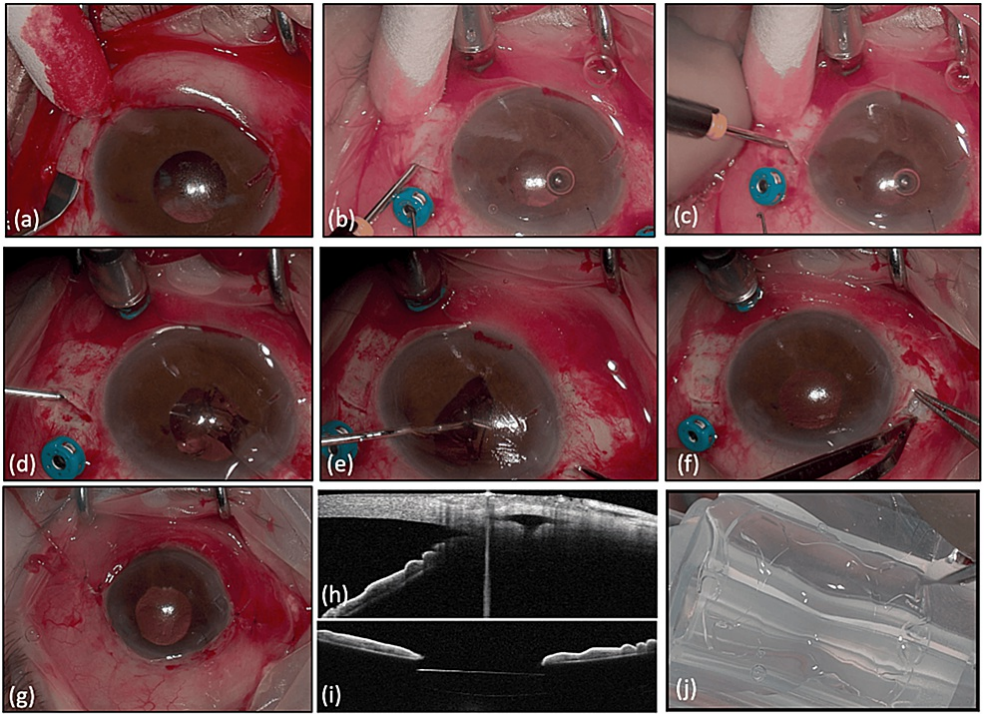
Surgical Technique and intraocular lens (IOL). The image shows the procedure steps and the final result - (a) creation of long scleral pocket, (b) a 23G trocar is inserted 2 mm inside the pocket and 1.7 mm away from limbus, (c) insertion of trocar with vertical angle inside the vitreous cavity, (d) inserting the folded lens from the main wound and simultaneously grasping the first plug with a forceps from the left sclerotomy, (e) grasping the second plug with a forceps from the left sclerotomy with handshake technique, (f) converting the right pocket into a triagonal shaped scleral flap and inspecting the position of the plug, (g) and at the end of the operation the conjunctiva is sutured with 7.0 Vicryl suture; (h) anterior chamber OCT confirming the intrascleral position of the plug, (i) anterior chamber OCT showing the lens behind the iris in proper position, and (j) preparation of Soleko lens for folding. OCT: optical coherence tomography

Prior to the surgery, retrobulbar anesthesia was administered in order to immobilize the eye. Conjunctival limbal dissection at three and nine clock hours, about 6 mm on each side was the first step of the surgery. Two, 4 mm long and 2.5 mm wide opposite scleral pockets, starting 1 mm and ending 3.5 mm behind the limbus, were created at three and nine clock hours, respectively (Figure 1, panel a). Then 25G standard pars plana vitrectomy (PPV) was performed using CONSTELLATION Vision System (Geneva, Switzerland: Alcon Inc.). The subluxated or dislocated IOL was introduced in the AC, cut and then removed from a 2.4 mm corneal incision. A 23G trocar with the cannula (21G in total) was inserted, parallel to the iris plane into the pocket sliding under the sclera until the middle of the pocket (2 mm) and 1.7 mm behind the limbus (Figure 1, panel b). Then the trocar punctured the sclera as perpendicularly as possible leaving the microcannula in place (Figure 1, panel c). The same scleral incision was made to the opposite scleral pocket.

The FIL SSF IOL was folded and then inserted through the pre-existing corneal incision (Figure 1, panel j). Then, with the handshake technique, using 25G non-serrated forceps, the plugs were externalized with caution from the scleral incisions remaining inside the pocket until only the edge of the plug was visible outside of the pocket (Figure 1, panel d). Using the same forceps both plugs were pushed inside the pockets until they reached the proper position (Figure 1, panel e). As a result, both plugs were completely covered by a partial thickness scleral layer inside a closed pocket which is essential for avoiding plug extrusion and improved scaring.

In two cases, during plug pushback, one plug fell back into the vitreous cavity. This happened because the plug was rotated and incompletely externalized through the sclerotomy. In order to ensure proper positioning of the plug, the surgeon converted the pocket into a trigonal-shaped scleral flap by making a 4 mm scleral incision parallel to the limbus at the margin of the pocket (Figure 1, panel f). The surgeon externalized the plug from the sclerotomy and confirmed its proper position before covering it with the scleral flap.

In order to avoid potential early post-operative hypotony, the opening of the pockets (or the edge of the flaps) was sutured with 7.0 Vicryl. The conjunctiva was sutured with 7.0 Vicryl at the end of the operation (Figure 1, panel g).

Results

In all cases, the lens was centered, not tilted, and not in contact with the iris as confirmed by anterior segment OCT (Figure 1, panel i). The lens haptics were in proper position covered by a continuous sclera layer. This finding was also confirmed by an anterior segment OCT (Figure 1, panel h). The refractive outcome was within the expectations according to biometry. The mean corrected post-operative visual acuity was 0.05±0.05 logMAR (range: 0.1-0 logMAR). The IOP was within normal limits.

## Discussion

The FIL SSF lens is specially designed for scleral fixation. With its novel design, each haptic has a special configuration providing elasticity and stability. Thus, it can remain in a centered position without interfering with other structures such as the iris. For this reason, it has less potential side effects than previously reported with other options such as the iris claw lenses (e.g., cystoid macular edema, pigment dispersion, iridodonesis, blurred vision when leaning forward) [8-11]. This option can also be considered superior to an intrascleral fixation of a three-piece IOL due to less post-operative astigmatism and reduced tilting or dislocation and bleeding [11,12]. Another advantage is that there is an option for a toric FIL SSF lens which can provide an even better refractive outcome.

Regarding the technique, when the FIL SSF lenses were first introduced, the most common implantation technique was the creation of two squared 4 mm flaps on opposite sides [4,5]. This straightforward technique was both safe and effective because surgeons could be sure of the proper position of the plugs and that they would always be covered by scleral layer. On the other hand, this technique is time-consuming and more traumatic. Our described technique is faster and less traumatic. In case a flap conversion is needed, this flap is triangular shaped instead of squared, thus the healing process is better and faster.

An alternative technique comprises the creation of four scleral pockets (two on each side), accommodating the lens plugs [4,6]. This technique is faster and easier than the regular flaps. However, there are some drawbacks regarding the wound structure of this option. The apex of the lens haptic (the anchor-shaped plug) exerts its force in the area between the pockets which is the most vulnerable site. This can potentially cause the plug to protrude. This might be the reason for the reported post-operative plug exposure [13]. From the author’s experience, another issue to take into account is that in some cases the sclera may be thin or the scleral tissue may be cut off during the process of pocket creation, due to the surgeon’s maneuvers. In this scenario, it will be difficult to cover the haptic, and perhaps a scleral transplant will be needed to cover the plug or create new pockets in different positions. With our proposed technique, the long scleral flaps provide a continuous scleral layer covering the plugs from all sides. In case of involuntary cutting of the scleral pocket tissue, this will have enough size to be immobilized with sutures, covering the hole plug. Our proposed technique of pocket creation can be considered more convenient and slightly faster than the other pocket creation technique because the pockets are created in one direction, instead of two opposite directions.

A similar, horizontal scleral pocket, technique has previously been described in a 2022 study [14]. In this technique, two side pockets (one nasal and one temporal) are created with a 2.4 mm incision parallel (instead of vertical) to the limbus. Then, sclerotomies are created by introducing the trocar ''blindly'' with a vertical angle to the limbus (posterior to anterior). In our opinion, this way of entering the vitreous cavity can be dangerous with the risk of damaging the ciliary body (instead we prefer to make the sclerotomy with an angle parallel to the limbus, as described above). Finally, in their technique, the plugs are then externalized blindly under the pockets and left inside them [14].

Until today there is at least one reported case of conjunctival erosion from lens plugs [12]. Even though there are no reports of endophthalmitis from the FIL SSF lens, it remains unclear whether a protruding plug under the conjunctiva can potentially cause endophthalmitis in the long term. The same consideration can be applied to the recently described transconjunctival technique, which is obviously the fastest and least traumatic but with higher risk, especially in elderly patients with little Tenon [7]. 

Another advantage of our described technique is that it is more versatile. As previously described, a surgeon can convert the long pocket into a scleral flap (Figure 1, panel f) at any time of the operation; thus, has better control of the process and modify his technique if needed. 

Our technique has some disadvantages. Sometimes, during the forceps insertion in the sclerotomy, localization of the sclerotomy under the pocket can be difficult. For this, surgeon can leave the trocars in the sclerotomy for localization and remove them during the haptic externalization. This also makes the sclerotomy wider (21G) reducing the risk of plug rupture. Moreover, during the externalization of the plugs, caution is needed in order to avoid exerting too much stress on the plugs due to the angulation generated inside the pocket. Nevertheless, these plugs have enough elasticity and we have not noticed any case of rupturing of the plug during externalization. Another disadvantage is that this technique was not tested with 25G trocars for the sclerotomy creation in conjunction with 27G forceps. This gauge could offer better recovery and more watertight wounds [13]. The rationale behind this choice is rooted in the surgeon's preference for the 23G sclerotomy. This preference is attributed to the wider nature of the 23G sclerotomy, which facilitates a safer externalization, makes it easier and more effective to push back the plugs into the pocket, and helps avoid the potential risks associated with plug rotation or angulation. Undoubtedly there are few patients recruited in this study, but the results are promising. The experience and the results provided by this study can justify a larger-scale comparative study of all techniques.

## Conclusions

We described a novel modified technique for FIL SSF lens (Rome, Italy: Soleko) implantation which has not been described before. This technique is faster and less traumatic than the squared flap technique and at the same time safer and perhaps easier than the previously described four-pocket technique. It is also versatile and convenient as it can be converted to a flap technique if needed. Larger prospective clinical trials could elucidate whether this is the most appropriate surgical implantation technique.
